# Why Another journal?

**DOI:** 10.4103/0974-7753.51913

**Published:** 2009

**Authors:** Patrick Yesudian

**Affiliations:** Department of Dermatology, Madras Medical College, No. 10 Ritherdon Avenue, Chennai - 600 007, India. Email: patnirmu@gmail.com


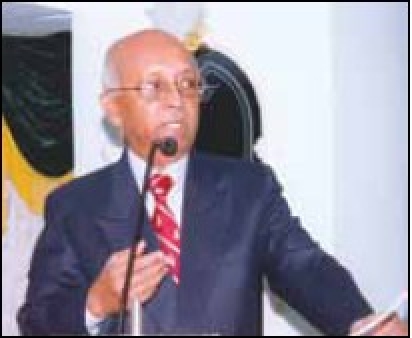


"Why another journal" may be a question asked by those seeing the *International Journal of Trichology*. A journal according to Webster′s dictionary is ′a periodical dealing especially with matters of current interest to a particular group′, in this instance dermatologists with a penchant for trichology. Interesting articles on trichology are often found in the dozens of dermatology journals in circulation but there is no journal dedicated solely to matters trichological. Since English being an international language of communication for trichologists, this journal is published in English to fill the long-awaited gap in this subspecialty of dermatology.

As a result of globalization at the turn of this century, a nova-like explosion was witnessed in technology, communication, and medicine. The field of trichology was no exception to these advances. The primitive ways of diagnosing and managing hair-related problems have been replaced by the use of the state-of-the-art equipments, techniques, and medication. Unfortunately, the fruits of such discoveries are yet to reach most of the developing countries who continue to live in the fringes of such advances. On the other hand, the clinical caseloads of interesting and exotic hair disorders are seen in developing countries of the world. This great divide can be bridged by a trichology journal, wherein cutting edge articles on recent advances published by research workers abroad will greatly benefit trichologists in developing nations, and conversely clinical trichology articles from here may be of interest to experts in developed countries.

*The International Journal of Trichology* is the official organ of the Hair Research Society of India, which was started four years ago with the aim of promoting ethical and scientific approach to hair disorders. With the current tendency for patients with hair disorders seeking the help of beauticians and unqualified cosmetologists and charlatans who make extraordinary and unrealistic claims using arbitrary treatment modalities, it was felt that dermatologists who are well versed in the pathophysiology of hair should take more interest in a scientific approach to hair disorders.

With this in mind, the Hair Research Society of India was started in a small way but has grown into a body of over 100 members with quarterly meetings and publication of newsletters. As a Chinese proverb says ′A journey of thousand miles start with a single step′, we have a long way to go but our efforts hitherto have culminated in the publication of the *International Journal of Trichology*. A glimpse of the members of the editorial board will show that the journal is indeed international. An international circulation of this journal would hopefully unite trichologists in the global village for the benefits of patients with hair disorders.

